# Temperature Compensation of Fiber Bragg Grating Sensors in Smart Strand

**DOI:** 10.3390/s22093282

**Published:** 2022-04-25

**Authors:** Se-Jin Jeon, Sung Yong Park, Sung Tae Kim

**Affiliations:** 1 Department of Civil Systems Engineering, Ajou University, 206, Worldcup-ro, Yeongtong-gu, Suwon-si 16499, Gyeonggi-do, Korea; 2 Department of Structural Engineering Research, Korea Institute of Civil Engineering and Building Technology, 283, Goyang-daero, Ilsanseo-gu, Goyang-si 10223, Gyeonggi-do, Korea; sypark@kict.re.kr (S.Y.P.); esper009@kict.re.kr (S.T.K.)

**Keywords:** prestressed concrete, prestressing tendon, strand, prestressing force, fiber optic sensor, fiber Bragg grating, FBG, temperature compensation, temperature correction, thermal sensitivity

## Abstract

Compared to other types of sensors, fiber optic sensors have improved accuracy and durability. Recently, the Smart Strand was developed to maximize the advantages of fiber optic sensors for measuring the cable forces in prestressed concrete structures or cable-supported bridges. The Smart Strand has fiber Bragg gratings (FBGs) embedded in a core wire of the seven-wire strand. Similar to other sensors, the strain measured at an FBG is affected by temperature; therefore, the temperature effect that is not related to the mechanical strain should be compensated for or corrected in the long-term measurement subjected to temperature variation. However, a temperature compensation procedure for the FBG has yet to be established, and relevant studies have used different formulas for the compensation. Moreover, when the FBG sensors are packaged with a certain material—such as fiber reinforced polymer—for protection, it is important to consider the interaction between the FBG, packaging material, and host material during thermal behavior. Therefore, this study proposed a reasonable procedure for temperature compensation for the FBG sensors embedded in packaging material and host material. In particular, the thermal sensitivity of the Smart Strand was intensively investigated. The proposed theoretical formulas were validated through comparison with data obtained from various specimens in a temperature-controlled chamber. Finally, the procedure was applied to correct the data measured using the Smart Strands in a 20-m-long full-scale specimen for about a year, thus resulting in a realistic trend of the long-term prestressing force.

## 1. Introduction

Conventional sensing technologies, such as approaches using electrical resistance strain gauges (ERSGs), have not been successfully applied for the continuous measurement of structural behavior for maintenance purposes due to their poor long-term performance resulting from their low durability. The lifetime of ERSGs is known to be much shorter than that expected of infrastructures [1].

On the other hand, proper estimation of the cable forces in prestressed concrete (PSC) structures or cable-supported bridges is a crucial factor for assessing structural safety and soundness during construction and while in service. A representative type of the cable used for these structures is the strand, such as a seven-wire strand [2]. Although there have been various attempts to measure the strand force by adopting various sensing technologies including ERSGs, it is difficult to accurately measure the complete force distribution along a strand. In addition to the above-mentioned durability problem, many of these approaches have several drawbacks and limitations: The load cell [3], lift-off test [4], and elasto-magnetic sensor [5] methods can only measure the strand force at the ends of a strand; the ultrasonic wave [6], vibration analysis [7], and acoustic emission [8] methods can only provide the averaged strand force along a strand rather than the strand force at a specific point; finally, ERSGs attached to the surface of the helical wires of a seven-wire strand are prone to damage during prestressing operation. Further, the strain of the helical wire cannot represent the true axial strain of a strand.

Fiber optic sensors are regarded as a promising solution due to their improved accuracy and durability compared to other sensors [9]. Among the different types of fiber optic sensors, fiber Bragg grating (FBG) has been preferred because it has a well-established theoretical background and there has been accumulated research on this type [10,11]. To maximize the advantages of the fiber optic sensor in applications intended to measure the strand force while overcoming the aforementioned drawbacks and limitations of the conventional sensors, the Smart Strand with FBG-type fiber optic sensors embedded in a core wire of the seven-wire strand was developed [12]. The Smart Strand has been applied to estimate the prestressing force (PF) distribution affected by the short-term prestress losses in full-scale PSC specimens and actual PSC structures [13,14,15,16]. The Smart Strand has also been used to investigate the time-dependent PF distribution caused by the long-term prestress losses [17]. Similar to other sensors, the strain measured at an FBG is affected by temperature; therefore, the temperature effect that is not related to the mechanical strain should be compensated for or corrected in the long-term measurement subjected to ambient temperature variation. However, many studies have performed temperature compensation (TC) or temperature correction for FBGs using an approximate formula or procedure without rigorous discussion [17,18,19,20,21].

In this respect, the TC procedure of the FBG has yet to be well established, and the relevant studies have shown some differences in the formulas they have used for the compensation. Moreover, when the FBG sensors are packaged with a certain material, such as the core wire of the Smart Strand, the interaction between the FBGs and the packaging material during thermal behavior should be considered in addition to the interaction between the FBGs and host material. Therefore, this study proposed a reasonable procedure of the TC for the FBG sensors embedded in a packaging material with a focus on the application to the Smart Strand. The proposed theoretical formulas were validated through comparison with the data obtained from various specimens in a temperature-controlled chamber. Finally, the procedure was applied to correct the data measured in a full-scale specimen for about a year.

## 2. Smart Strand with Embedded FBGs

**Figure 1:**
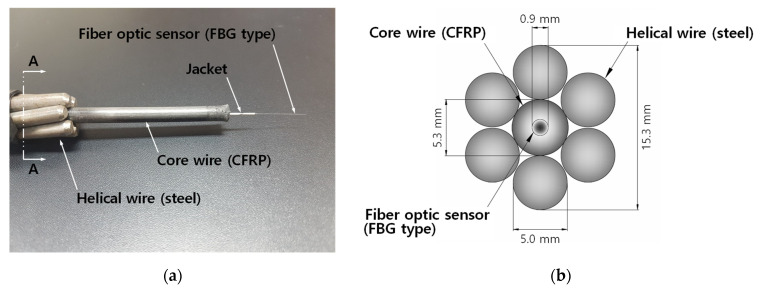
Configuration and dimensions of a Smart Strand: (**a**) configuration; and (**b**) section A–A and dimensions.

Figure 1 shows the configuration and main dimensions of a Smart Strand, which are almost identical to those of a regular seven-wire strand with ultimate tensile strength of 1860 MPa and a diameter of 15.24 mm [2]. However, the steel core wire in a regular strand is replaced with a carbon fiber reinforced polymer (CFRP) core wire to accommodate the embedment of a fiber optic sensor with a required number of FBGs while the CFRP core wire is manufactured through a pultrusion process. The fiber optic sensor surrounded by the CFRP core wire can be protected from the damage caused by the contact with a duct and adjacent strands during the prestressing operation, and it can measure the true axial strain of a strand. In addition to the sensor function, the Smart Strand also has a function of a structural component because the stress–strain relation of a Smart Strand is similar to that of a regular strand. More detailed information on the development of the Smart Strand can be found in the literature [12].

Although previous studies have attempted to embed FBGs into the steel or CFRP core wire of a strand [18,22,23], the Smart Strand in this study was subjected to more validation cases than those examined in the other studies through field measurement in actual structures and full-scale specimens [13,14,15,16,17].

Equation (1) is the basic formula used to convert the change in the wavelength of a light wave reflected at each FBG to the strain. Equation (1) can be extended to Equation (2) to include the term for TC that is required when the effect of ambient temperature on the strain is dominant, such as in the long-term measurement [24].

(1) $$\varepsilon_{m}=\frac{1}{1-p_{e}}\cdot \frac{\Delta \lambda }{\lambda_{B}},$$

(2) $$\varepsilon_{m}=\frac{1}{1-p_{e}}\left(\frac{\Delta \lambda }{\lambda_{B}}-K_{T}\Delta T\right),$$

where $\varepsilon_{m}$: mechanical strain, $p_{e}$: photo-elastic coefficient, $\Delta \lambda =\lambda -\lambda_{B}$: wavelength shift, λ: measured wavelength, $\lambda_{B}$: base wavelength at the start of measurement, $K_{T}$: thermal sensitivity, $\Delta T=T-T_{B}$: temperature change, T: measured temperature, and $T_{B}$: base temperature (also called reference temperature) at the start of measurement. The reasonable forms and values of $K_{T}$ in various cases, including the application to Smart Strand, were investigated in this study, as shown in later sections.

In the case of the Smart Strand, the strain obtained in Equations (1) or (2) can further be converted to PF by using the linear force–strain relation shown in Equation (3), which is practically valid in the service stage of PSC structures.

(3) $$P={\left(E_{p}A_{p}\right)}_{smart}\varepsilon_{p},$$

where P: PF at an FBG, ${\left(E_{p}A_{p}\right)}_{smart}$: equivalent $E_{p}A_{p}$ of a Smart Strand, $E_{p}$: modulus of elasticity of a strand, $A_{p}$: cross-sectional area of a strand, and $\varepsilon_{p}$: strain measured at an FBG of a Smart Strand, which corresponds to $\varepsilon_{m}$ in Equation (1) or (2). The value of ${\left(E_{p}A_{p}\right)}_{smart}$ was experimentally obtained as 26,600 kN in a tensile test of a Smart Strand, which was attributed to the fact that the Smart Strand is the hybrid material of a CFRP core wire and steel helical wires, as shown in Figure 1.

## 3. Formulas for TC

### 3.1. Basic Formula

The general relationship between the strains can be expressed by Equation (4).

(4) $$\varepsilon_{m}=\varepsilon -\varepsilon_{t},$$

where $\varepsilon_{m}$: mechanical strain, ε: total strain that is actually measured using a strain gauge (FBG, ERSG, etc.), and $\varepsilon_{t}$: thermal strain. The mechanical strain is of primary importance because it is directly related to the stress that is to be obtained for the purpose of structural analyses. Therefore, to obtain the mechanical strain, the thermal strain should be deducted from the measured total strain; this process is called TC. In the case of the FBG shown in Equation (2), $\left[1/\left(1-p_{e}\right)\right]\Delta \lambda /\lambda_{B}$ corresponds to the total strain, and $\left[1/\left(1-p_{e}\right)\right]K_{T}\Delta T$ to the thermal strain.

The thermal strain can largely be obtained in two ways: theoretical derivation or the use of dummy sensor. Dummy sensing is useful in validation of the theory because the theory may inevitably involve a few assumptions, and the values of the coefficients used in the calculation may not be sufficiently accurate in some cases. The dummy sensing can be explained as follows: if the strain is measured in a controlled circumstance that does not induce any mechanical strain, then the measured strain corresponds to the thermal strain itself. That is, given $\varepsilon_{m}$ = 0, $\varepsilon =\varepsilon_{t}$ in Equation (4). The controlled circumstance can be realized by separating a sensor from adjacent materials and by removing any external restraint for thermal expansion or contraction. It should be noted that, if the thermal deformation is restrained, the mechanical strain can even be induced by the temperature.

### 3.2. Formulas in Previous Studies

For an isolated FBG that has no interaction with the host material of which the strain is measured, Magne et al. [25] provided Equation (5).

(5) $$\frac{\Delta \lambda }{\lambda_{B}}=\left(1-p_{e}\right)\varepsilon_{fm}+\left(\alpha_{f}+\xi \right)\Delta T,$$

where $\varepsilon_{fm}$: mechanical strain of an FBG, $\alpha_{f}$: thermal expansion coefficient of an FBG, ξ: thermo-optic coefficient, and all other notations have the same meanings as in Equation (2). This means that $K_{T}=\alpha_{f}+\xi$ in Equation (2) in this isolated condition of an FBG. They suggested the following values for the coefficients: $p_{e}$ = 0.22, $\alpha_{f}$ = 0.5 × 10^−6^/°C, and ξ = 7 × 10^−6^/°C. They also provided Equation (6), without any derivation of the formula, which can be applied when an FBG is embedded into or attached onto a host material. Although they did not mention whether the mechanical strain in Equation (6) is for the FBG or for the host material, a later section in this paper will clarify that the formula is expressed for the mechanical strain of the host material.

(6) $$\varepsilon_{hm}=\frac{1}{1-p_{e}}\left\{\frac{\Delta \lambda }{\lambda_{B}}-\left[\alpha_{f}+\xi +\left(1-p_{e}\right)\left(\alpha_{h}-\alpha_{f}\right)\right]\Delta T\right\},$$

where $\varepsilon_{hm}$ and $\alpha_{h}$ are the mechanical strain and thermal expansion coefficient of host material, respectively. They also mentioned an approximate form of Equation (6) in the general condition of $\alpha_{h}\gg \alpha_{f}$, where $\alpha_{f}$ can be ignored as zero in Equation (6). This approximate formula was also presented by Pereira et al. [26], but they attributed the approximation to the difference in stiffness between FBG and the host material rather than that between different thermal expansion coefficients.

Kreuzer [27] provided a series of formulas for TC that differ from the aforementioned ones; however, these formulas do not appear to be generally accepted or used in the theory of FBG. He used the following values: $p_{e}$ = 0.22, $\alpha_{f}$ = 0.55 × 10^−6^/°C, and ξ = (5~8) × 10^−6^/°C.

Zhou and Ou [20] used a simpler form of Equation (7) than Equation (6) for the same situation; their reasoning was that the deformation of an FBG follows that of the host material, which in their study was cement paste. They used $p_{e}$ = 0.22 and $\alpha_{f}+\xi$ = 6.67 × 10^−6^/°C. Therefore, if we assume $\alpha_{f}$ = 0.5 × 10^−6^/°C, ξ = 6.17 × 10^−6^/°C can be obtained.

(7) $$\varepsilon_{hm}=\frac{1}{1-p_{e}}\left[\frac{\Delta \lambda }{\lambda_{B}}-\left(\alpha_{h}+\xi \right)\Delta T\right].$$

Kim et al. [18,19] developed a strand with the FBGs encapsulated into a steel core wire, but they simply assumed that $\varepsilon_{hm}$ can be substituted for $\varepsilon_{fm}$ in Equation (5) without a strict discussion of this assumption. They also mentioned that $\alpha_{f}$ in Equation (5) can be replaced with $\alpha_{h}$ due to the composite action between the FBG and the host material. As a result, they used the same formula as Equation (7). Although it is apparent that the host material in this case is the strand and not concrete, they misused the $\alpha_{h}$ of concrete instead of that of the strand. They used $p_{e}$ = 0.22, $\alpha_{f}$ = 0.55 × 10^−6^/°C, and two ξ values: ξ = 8.6 × 10^−6^/°C as a textbook value and ξ = 5.67 × 10^−6^/°C as their experimentally obtained value. Kim et al. [17] took a similar approach to that of Kim et al. [18,19] in dealing with the TC of the same Smart Strand as that used in this study.

### 3.3. Derivation of Reasonable Formulas for TC

As discussed in Section 3.2, previous studies have used different formulas for the mechanical strain of a host material. Moreover, there has not been a consistent and reasonable discussion on the general form of the formulas for TC in the case of multiple host materials. Therefore, the main purpose of this study is to address these problems both theoretically and experimentally.

If the variation of temperature is not considered in a single measurement, as opposed to a continuous long-term measurement, then there is no complicated problem because the mechanical strain is the measured total strain itself and the mechanical strain of FBG coincides with that of the host materials due to the composite action.

It should be noted that the strains that we aim to obtain are those of the host material, not those of FBGs themselves, in consideration of the purpose of sensing in the present work to examine the behavior of a structural component. Therefore, for the example of a PSC structure, the host material can be anything between concrete, reinforcements, and prestressing tendons. In some cases, a host material can further be divided into sub-host materials, such as in the Smart Strand, which consists of a CFRP core wire and six steel helical wires, thus indicating the presence of two types of host materials.

For the purpose of convenience in terms of usage and mechanical protection, FBGs are typically encapsulated into another material such as FRP or metal. Then, this packaging material with the FBGs inside can be embedded into a host material (strand), as has been done in other studies [18,23] and this study [12], as shown in Figure 1, or embedded into a host material (concrete) [20,21], or attached onto a host material (strand) [28,29]. However, the packaging material and host material do not have to be distinguished in terms of the formulas for TC; therefore, they will commonly be called host material hereafter.

**Figure 2:**
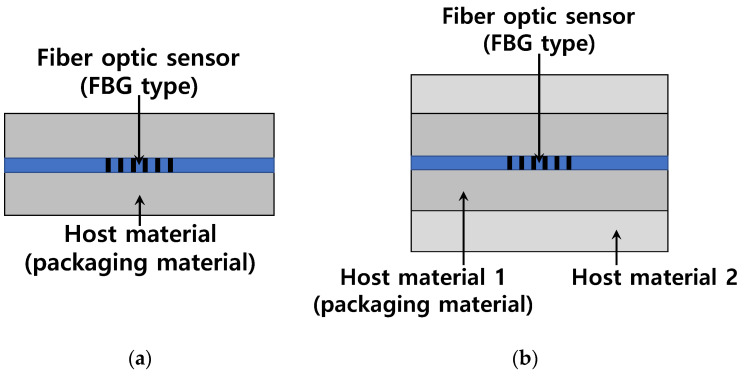
FBG embedded in host materials: (**a**) one host material; and (**b**) two host materials.

Let us start with the case of a single host material as illustrated in Figure 2a. The total strains of the FBG and the adjacent host material can be respectively expressed by Equations (8) and (9) after modifying Equation (4).

(8) $$\varepsilon_{f}=\varepsilon_{fm}+\varepsilon_{ft},$$

(9) $$\varepsilon_{h}=\varepsilon_{hm}+\varepsilon_{ht},$$

where the subscripts f and h represent FBG (or fiber optic sensor) and the host material, respectively, while the subscripts m and t indicate mechanical strain and thermal strain, respectively, as was the case in Equation (4). There is broad consensus for accepting Equation (5) as a formula for the mechanical strain of the FBG that is separated from a host material, and the thermal strains are as follows: $\varepsilon_{ft}=\alpha_{f}\Delta T$ and $\varepsilon_{ht}=\alpha_{h}\Delta T$. If the FBG exhibits composite action with the host material, thereby satisfying the strain compatibility of $\varepsilon_{f}=\varepsilon_{h}$, Equation (6) can be derived from the equality of $\varepsilon_{hm}=\varepsilon_{fm}+\varepsilon_{ft}-\varepsilon_{ht}$ by substituting the above-mentioned related formulas. As a result, the validity of Equation (6) can be theoretically confirmed, whereas it was revealed that Equation (7), in comparison to the exact expression of Equation (6), shows a lack of a reasonable theoretical basis regardless of the amount of errors.

The discussion can be extended to cover the multiple host materials used. Figure 2b shows the case of two host materials. Provided that the FBG and all the host materials show composite action through perfect bond or embedment, the strain compatibility can also be established between the FBG and the host material 2. Equation (6) can therefore be generalized to express the relationship between an FBG and any host material n in not less than n multiple host materials, as shown in Equation (10), where the subscript n represents an identification number of the host material of concern.

(10) $$\varepsilon_{hnm}=\frac{1}{1-p_{e}}\left\{\frac{\Delta \lambda }{\lambda_{B}}-\left[\alpha_{f}+\xi +\left(1-p_{e}\right)\left(\alpha_{hn}-\alpha_{f}\right)\right]\Delta T\right\}.$$

### 3.4. Formulas for Smart Strand

According to the above derivation, the mechanical strains of a core wire and helical wires, which are $\varepsilon_{cm}$ and $\varepsilon_{hm}$, respectively, can be obtained by substituting $\alpha_{c}$ and $\alpha_{h}$ for $\alpha_{hn}$ in Equation (10), respectively. The subscripts c and h were used for the core wire and helical wires, respectively. This leads to a question regarding the true mechanical strain of the Smart Strand, because the values of $\varepsilon_{cm}$ and $\varepsilon_{hm}$ are different due to the difference between $\alpha_{c}$ and $\alpha_{h}$.

One noteworthy aspect in the Smart Strand is that the CFRP core wire can be regarded as both a packaging material and a host material, while the steel helical wires can be considered another host material. Between the FBG (also called core) and the CFRP, there are a series of very thin layers of cladding, coating, and jacket. However, the stiffness of these in-between materials is so small that it has a negligible effect on the thermal behavior of the Smart Strand.

**Figure 3:**
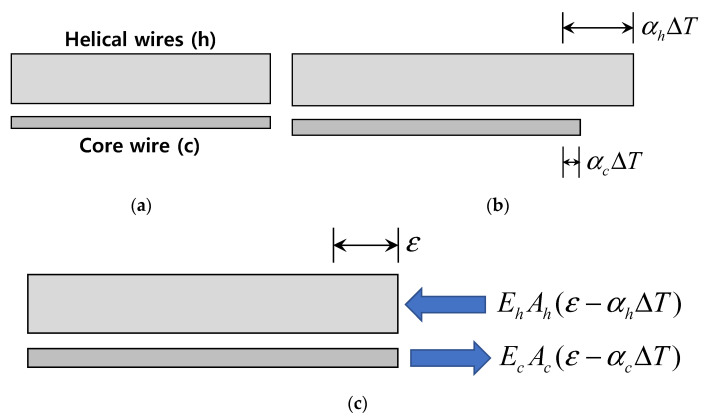
Thermal behavior of a core wire and helical wires in a Smart Strand: (**a**) before the length change; (**b**) length change without restraint; and (**c**) length change with restraint (actual situation).

The most comprehensive approach for deriving the mechanical strain of the Smart Strand is to consider the composite action and mutual restraint of a CFRP core wire and steel helical wires. That is, the core wire and helical wires should be considered together as another integrated host material, after which, the equivalent thermal expansion coefficient of this integrated Smart Strand can be derived. Figure 3b shows the thermal change of the lengths in a core wire and helical wires without mutual restraint from the original shape in Figure 3a. However, because the core wire and helical wires are tightly bound together when manufacturing a Smart Strand, the final length of these two materials should be the same, as shown in Figure 3c. Consequently, the compressive or tensile forces shown in Figure 3c are induced in the helical wires and core wire, respectively, to satisfy the strain compatibility, and Equation (11) can be derived from the self-equilibrium of these two forces.

(11) $$\varepsilon =\frac{\alpha_{c}E_{c}A_{c}+\alpha_{h}E_{h}A_{h}}{E_{c}A_{c}+E_{h}A_{h}}\Delta T=\alpha_{smart}\Delta T,$$

where E: modulus of elasticity and A: cross-sectional area, with the subscripts c and h respectively representing a core wire and helical wires, and $\alpha_{smart}$: equivalent thermal expansion coefficient of a Smart Strand. $\alpha_{smart}$ = 8.68 × 10^−6^/°C can be obtained by substituting the following values in Equation (11) based on the configuration of the Smart Strand in Figure 1 and the general material properties of CFRP and steel: $\alpha_{c}$ = 0, $E_{c}$ = 1.63 × 10^5^ MPa, $A_{c}$ = 22.06 mm^2^, $\alpha_{h}$ = 10 × 10^−6^/°C, $E_{h}$ = 2 × 10^5^ MPa, and $A_{h}$ = 117.78 mm^2^. $\alpha_{c}$ = 0 was used in this calculation despite the fact that $\alpha_{c}$ ranges from −1 × 10^−6^/°C to zero depending on the fiber-volume fraction [30]. Even if an extreme value of $\alpha_{c}$ = −1 is assumed, $\alpha_{smart}$ results in 8.54 × 10^−6^/°C with a difference of only 1.6% from that for $\alpha_{c}$ = 0. It can be seen that the axial stiffness and thermal expansion coefficient of the steel helical wires are more dominant than those of a CFRP core wire, resulting in a small difference of 13% from $\alpha_{h}$. However, the contribution of the core wire is still significant in $\alpha_{smart}$, so it should not be ignored.

Therefore, the mechanical strain of the Smart Strand can be represented by Equation (12).

(12) $$\varepsilon_{smart,m}=\frac{1}{1-p_{e}}\left\{\frac{\Delta \lambda }{\lambda_{B}}-\left[\alpha_{f}+\xi +\left(1-p_{e}\right)\left(\alpha_{smart}-\alpha_{f}\right)\right]\Delta T\right\}=\frac{1}{1-p_{e}}\left(\frac{\Delta \lambda }{\lambda_{B}}-K_{T,smart}\Delta T\right),$$

where $K_{T,smart}$: thermal sensitivity of a Smart Strand. Table 1 presents a comparison of the forms of $K_{T,smart}$. The values of several coefficients were adopted from manufacturers’ specifications and the previous studies referenced in Section 3.2. The approximate formula 1 is an approach to ignore $a_{f}$ in the exact formula, as was mentioned in Section 3.2 and in the referenced papers [25,26]. The approximate formula 2 has been used in some previous studies [17,19,20] without a strict theoretical basis, as described in Section 3.2. The approximate formula 1 is also acceptable because it shows only 0.8% error when compared to the exact formula, whereas the approximate formula 2 shows an error of as much as 23.7%, which is attributed to the significant and unacceptable approximations.

As alternatives to the Smart Strand, we briefly discuss herein how to deal with other types of application of the FBG sensor. When the FBG packaged with a short-length of CFRP or another material is embedded into a large-sized host material such as a concrete member [20,21] or attached onto a strand [28,29] or a rebar, the axial stiffness of the host material is much more dominant than that of the packaging material. Then, Equation (10) can simply be applied without considering the interaction between the packaging material and the host material. That is, the contribution of the packaging material in the TC can be ignored in these applications.

## 4. Chamber Test for Validation of Thermal Sensitivity

### 4.1. Test Setup

As was discussed in Section 3.1, $K_{T,smart}$ in Equation (12) can be validated by preparing the circumstances for dummy sensing, thus eliminating any stress-inducing conditions. That is, Equation (13) can be established by substituting $\varepsilon_{smart,m}$ = 0 in Equation (12).

(13) $$\frac{\Delta \lambda }{\lambda_{B}}=K_{T,smart}\Delta T.$$

Because this principle can be applied to any type of specimen, several types of specimens were tested in addition to the Smart Strand specimen to also examine the validity of the coefficients included in the formula for TC.

**Figure 4:**
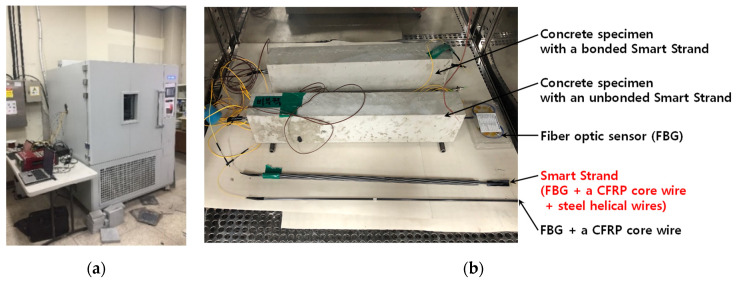
Chamber test: (**a**) temperature-controlled chamber; and (**b**) various specimens.

Figure 4 shows the temperature-controlled chamber with a space of 900 × 900 × 900 mm used for testing; several specimens are placed inside. All the specimens had the length of 600 mm and an FBG was located at the middle. Although two concrete specimens with a bonded or an unbonded Smart Strand were also tested, they are beyond the scope of this study and are not analyzed herein. To avoid any frictional restraint for thermal deformation, a Teflon sheet with a smooth surface was laid below the specimens. A few thermocouples were placed adjacent to the specimens to measure the actual temperature for compensation. Emission of light and data acquisition of the light waves reflected at FBGs were performed using an optical interrogator.

**Figure 5:**
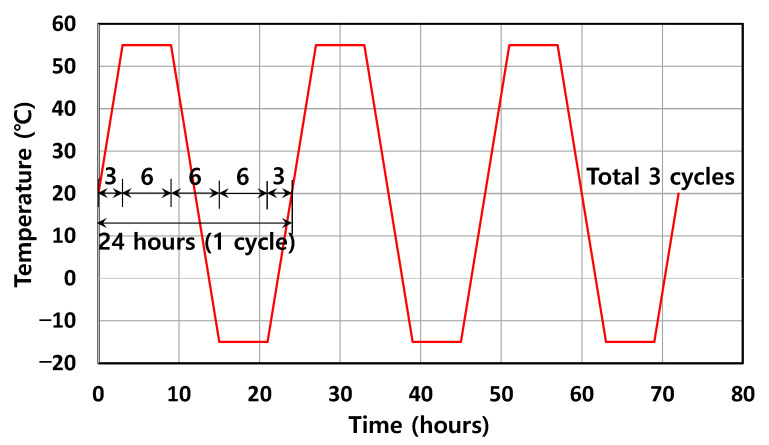
Temperature history inside the chamber.

As shown in Figure 5, the chamber temperature was cyclically varied in a sufficient range from −15 °C to 55 °C—which means ΔT = 70 °C or 20 °C (room temperature) ± 35 °C—to minimize the error in the validation of Equation (13) by increasing the response of the wavelength. The maximum and minimum temperatures were maintained for 6 h, and the in-between temperature was gradually varied for 6 h to provide sufficient time for the specimens to attain the chamber temperature. A total of three cycles were applied, with each cycle being 24 h in length.

### 4.2. Test Results

#### 4.2.1. Fiber Optic Sensor (FBG)

**Figure 6:**
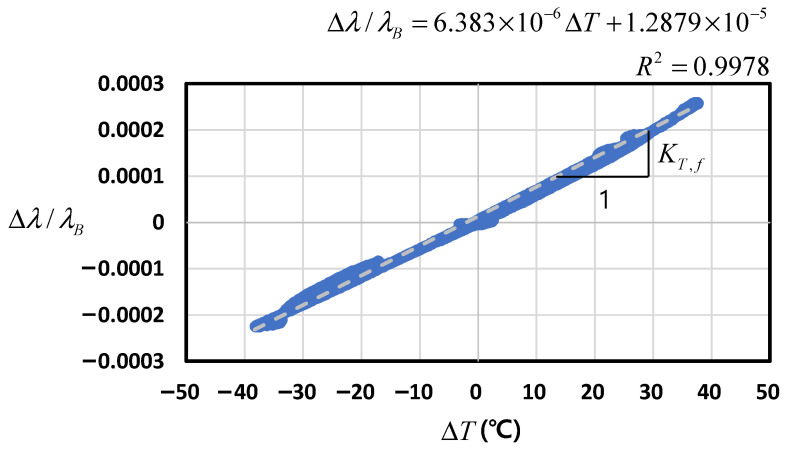
Wavelength–temperature relation of FBG.

After modifying Equation (5) to represent the dummy sensing condition by setting $\varepsilon_{fm}$ = 0, the thermal sensitivity for FBG ($K_{T,f}$) equals $\alpha_{f}+\xi$. The initial values were measured as $T_{B}$ = 20.1°C and $\lambda_{B}$ = 1520.00066 nm. Figure 6 shows the measured wavelength according to the cyclic temperature variation, where the slope corresponds to $K_{T,f}$. The linear regression equation denoted by a dashed line and the coefficient of determination ($R^{2}$) are also presented in Figure 6. Because $R^{2}$ approached unity, the regression equation shows a correlation that is highly statistically significant. As a result, $K_{T,f}$ = 6.4 × 10^−6^/°C was obtained. Therefore, if we assume $\alpha_{f}$ = 0.5 × 10^−6^/°C, then ξ can be estimated as 5.9 × 10^−6^/°C, which is similar to the value assumed in Table 1. In this method, the values of the various coefficients can be validated and corrected through the chamber test if necessary.

#### 4.2.2. Fiber Optic Sensor (FBG) + CFRP Core Wire

**Figure 7:**
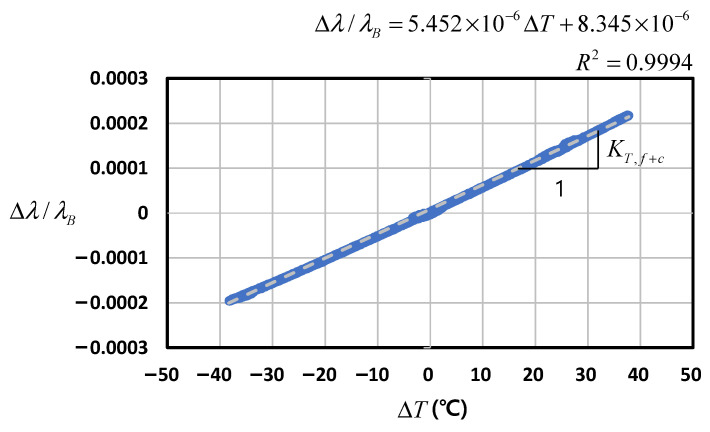
Wavelength–temperature relation of FBG + CFRP core wire.

The relevant formula is Equation (6), where the host material in this case is the CFRP core wire. Because the axial stiffness of the core wire is much more dominant than that of the fiber optic sensor, almost no mechanical strain of the core wire is induced by the restraint of the fiber optic sensor when the specimen deforms axially during temperature variation. The corresponding thermal sensitivity ($K_{T,f+c}$) is $\alpha_{f}+\xi +\left(1-p_{e}\right)\left(\alpha_{c}-\alpha_{f}\right)$. With the initial values of $T_{B}$ = 20.1°C and $\lambda_{B}$ = 1550.44846 nm, the relation shown in Figure 7 and $K_{T,f+c}$ = 5.5 × 10^−6^/°C were obtained. Given $\alpha_{f}$ = 0.5 × 10^−6^/°C, $p_{e}$ = 0.22, and $\alpha_{f}+\xi$ = 6.4 × 10^−6^/°C from the FBG test described in Section 4.2.1, $\alpha_{c}$ can be estimated as −0.65 × 10^−6^/°C, which falls within the general range of CFRP discussed in Section 3.4.

#### 4.2.3. Smart Strand: Fiber Optic Sensor (FBG) + CFRP Core Wire + Steel Helical Wires

**Figure 8:**
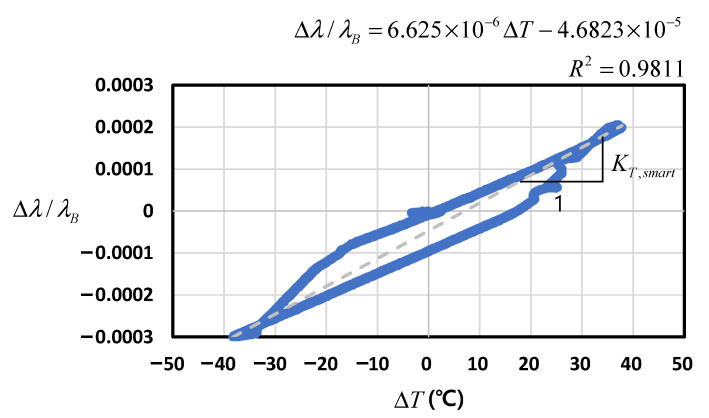
Wavelength–temperature relation of Smart Strand.

The thermal sensitivity of the Smart Strand ($K_{T,smart}$ or, alternatively, $K_{T,f+c+h}$) in Equation (13) was investigated. Figure 8 shows the measured wavelength-temperature relation of the Smart Strand with the initial values of $T_{B}$ = 20.1°C and $\lambda_{B}$ = 1550.37142 nm.

The graphs in Figure 8 did not show a linear trend passing through the origin, unlike those in Figure 6 and Figure 7. Therefore, although a linear regression equation was plotted, resulting in $K_{T,smart}$ = 6.6 × 10^−6^/°C, the results could not be considered accurate and reliable. The $K_{T,smart}$ was much smaller than the theoretically obtained value of 13.1 × 10^−6^/°C presented in Table 1. The main reason for this phenomenon seems to be the unanticipated non-composite action between the core wire and the helical wires in the Smart Strand specimen. As mentioned in Section 4.1, the longitudinal length of the specimen was 600 mm, which was set in consideration of the chamber size. However, the length was not long enough to realize tight binding of the core wire and helical wires; therefore, slip appeared to occur at the interface of the wires. This means that the assumption of the perfectly composite action made in Figure 3 to derive Equation (12) is no longer valid. In an actual PSC structure, both ends of a strand are anchored at the anchor heads using the wedges, where the seven wires are tightly bound together, while the ends of the Smart Strand specimen remained untreated in the test in the present work. Meanwhile, the Smart Strand specimen tested in an earlier study [31] had a longer length of 1000~1500 mm than that in this study, thus providing larger contact area between the wires, and it resulted in $K_{T,smart}$ = 12.5 × 10^−6^/°C, which is similar to the theoretical value.

In summary, it was revealed that the specimen of a Smart Strand used in the test of TC should be long enough to maintain a high degree of restraint between the wires during longitudinal thermal deformation. Alternatively, if the specimen length is not sufficiently long, both ends of the Smart Strand specimen should be tightly bound to ensure that the changes in the longitudinal length are the same between the wires.

#### 4.2.4. Comparison between Theory and Experiment

Various theoretical and experimental values of thermal sensitivity are compared in Table 2. For the FBG sensor in this test, $K_{T,f}$ was closer to the theory—with a difference of 4.5%—than that in the previous test. $K_{T,f+c}$ for the FBG sensor encapsulated into a CFRP core was identical in the previous and current tests, and it was smaller than the theoretical value by 12.7%. Although $K_{T,f}$ and $K_{T,f+c}$ showed some differences from the theoretical values, the tendency of $K_{T,f}>K_{T,f+c}$ was identified in both the previous and current tests, in accordance with the theory.

However, $K_{T,smart}$ or $K_{T,f+c+h}$ measured in this test showed significant differences from the theoretical value for the reasons stated in Section 4.2.3. Instead, the $K_{T,smart}$ obtained in the previous test [31] exhibited a plausible value with only a 4.6% difference from the theory. Meanwhile, the theoretical $K_{T,smart}$ derived in this study is reliable because the values of the coefficients included in the formula of $K_{T,smart}$ were validated to some extent through the chamber test described in Section 4.2.1 and 4.2.2.

## 5. Application to a Post-Tensioned Full-Scale Specimen

### 5.1. Fabrication of Specimen

**Figure 9:**
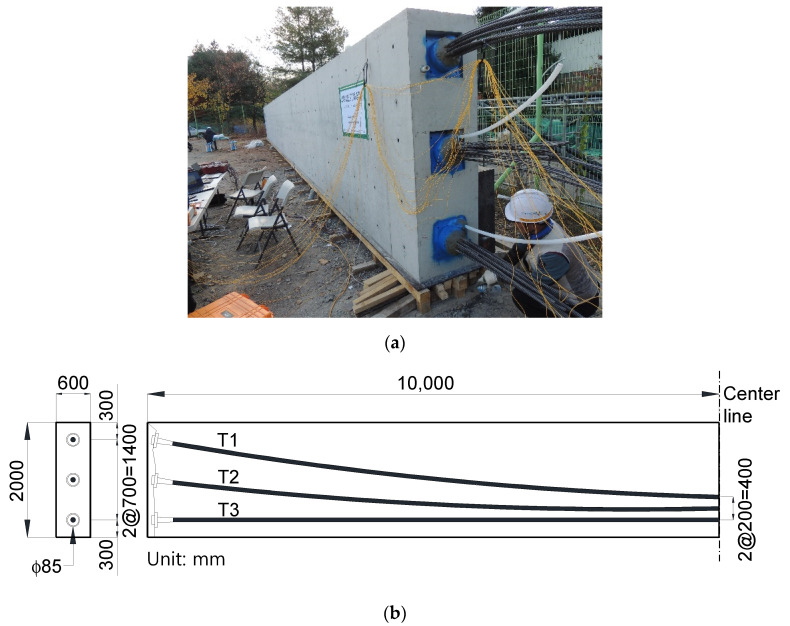
Post-tensioned full-scale specimen: (**a**) overall view; and (**b**) dimensions.

To investigate the short- and long-term characteristics of PF related to various prestress losses using Smart Strands, a 20-m-long post-tensioned full-scale specimen was fabricated and exposed to ambient temperature for 318 days, as shown in Figure 9. More detailed information and analysis results of this specimen can be found in previous studies [16,17]. Three ducts with a diameter of 85 mm—denoted by T1, T2, and T3—were arranged with 12 strands inserted in each duct. The Smart Strands shown in Figure 1 were selectively inserted together with regular strands. Three types of the Smart Strands were fabricated with three, five, and seven FBGs along the strand, respectively. The regular strand has a diameter of 15.2 mm and an ultimate tensile strength of 1860 MPa. All the strands were tensioned up to 70% of 1860 MPa, with PF of 180 kN introduced in each strand, when the concrete compressive strength attained 30 MPa. After the tensioning and anchoring of the strands, all the ducts were grouted.

### 5.2. Effect of TC on Long-Term PF

**Figure 10:**
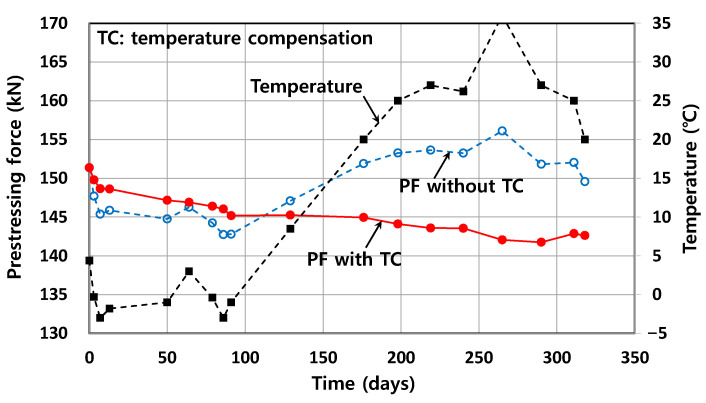
Effect of TC on PF.

Figure 10 shows the variations of temperature and PF measured at an FBG located at the mid-span of a Smart Strand inserted in T1. The temperature was measured at a thermocouple located near the FBG with the aim of performing more accurate TC than that using the ambient temperature. The PF can be obtained from the wavelength of an FBG by applying the conversion formulas of Equations (2) and (3). Obviously, during the long-term measurement, the strains and PFs measured by sensors are affected by seasonal and daily variations in temperature. According to the theory of prestress losses, the PF gradually decreases in the long-term due to the creep and shrinkage of concrete and the relaxation of a strand. However, Figure 10 shows that the regular trend of such a long-term PF distribution can only be achieved through a reasonable TC, where $K_{T,smart}$ = 13.1 × 10^−6^/°C in Table 1 was applied. Unless the TC is conducted, the PF was abnormally fluctuated by the effect of the temperature variation.

Note that the PF distribution compensated for the temperature can even be slightly fluctuated with time in a special case when the strand is lengthened or shortened by the deformation of the concrete member subjected to temperature variation. However, this effect is not seen in Figure 10 because the thermal expansion coefficient of concrete (which is typically 10 × 10^−6^/°C) is similar to that of the strands including Smart Strands. That is, in this case, temperature-induced “mechanical” strains of the strands were negligible.

**Figure 11:**
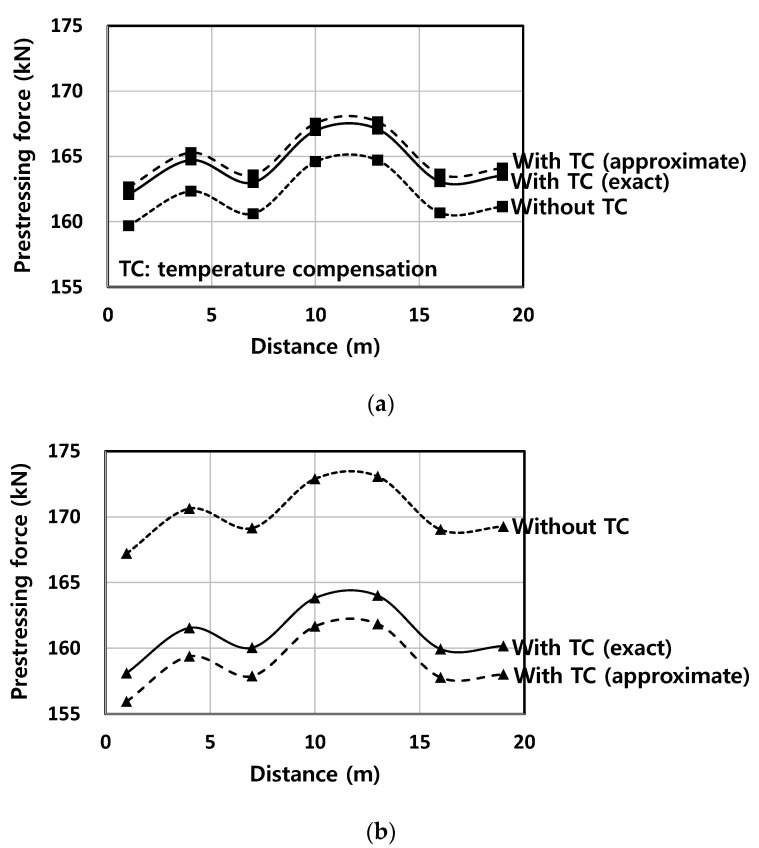
Effect of TC on PF distribution: (**a**) 91 days after tensioning; and (**b**) 198 days after tensioning.

Figure 11 shows the PF distribution along another Smart Strand inserted in T1 at two time points, which was obtained by connecting the PF values measured at the FBGs that were represented by the markers. The exact TC corresponds to $K_{T,smart}$ = 13.1 × 10^−6^/°C whereas the approximate TC is indicated as “Approximate 2” with $K_{T,smart}$ = 16.2 × 10^−6^/°C in Table 1. “Approximate 1” in Table 1 was excluded from the analysis because it has an almost identical value to the exact one.

The temperature changes (ΔT) after tensioning were −5.4 °C and 20.6 °C for 91 days and 198 days, respectively, in Figure 11. The errors caused by non-compensation of the temperature were 1.43~1.47% and 5.53~5.76%, and those between the exact compensation and the approximate one were 0.34~0.35% and 1.31~1.37% for Figure 11a,b, respectively. Therefore, these two types of errors were increased as the ΔT increased. In particular, when ΔT is large, a large error can be caused by omitting the TC. It can also be seen from Figure 11 that the approximate thermal sensitivity can over-compensate or under-compensate the temperature, thus leading to unreliable PF distribution depending on the magnitude of ΔT. Therefore, when compensating the temperature for FBG, it is crucial to derive and apply the accurate thermal sensitivity with a reasonable theoretical basis and sufficient experimental validation.

**Figure 12:**
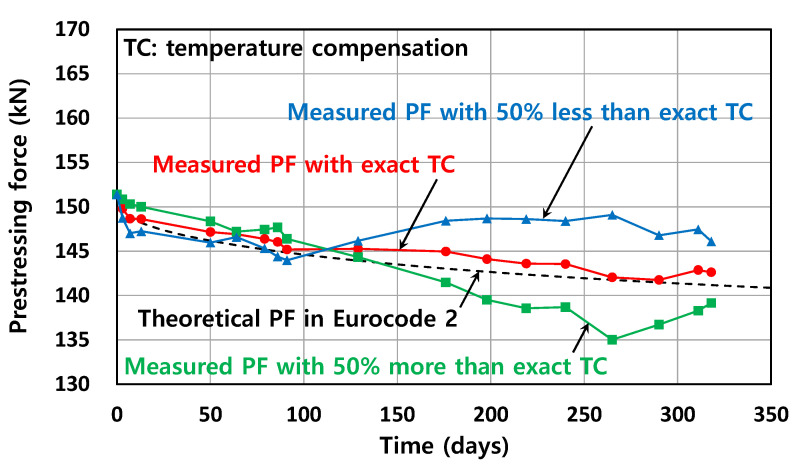
Comparison of PFs at mid-span for various TC.

Figure 12 compares the PF of the Smart Strand, which was temperature-compensated as shown in Figure 10, with the theoretical PF obtained from the design formula of Equation (14). Figure 12 also shows PFs intentionally over-compensated or under-compensated by 50% relative to the exact $K_{T,smart}$ to demonstrate the effect of the inaccurate TC on the PFs. Equation (14) accommodates the calculation of long-term prestress losses and is specified in Eurocode 2 [32], which is one of the representative design codes for concrete structures. The theoretical PF can be obtained by subtracting the PF loss, which is the prestress loss multiplied by the area of a strand, from the initial PF before the losses.

(14) $$\Delta f_{p,CR+SH+R}=\frac{E_{p}{\left(\varepsilon_{sh}\right)}_{t}+0.8\Delta f_{pR}+nC_{t}f_{c}}{1+n\frac{A_{p}}{A_{c}}\left(1+\frac{A_{c}}{I_{c}}e_{p}{}^{2}\right)\left(1+0.8C_{t}\right)},$$

where $E_{p}$: modulus of elasticity of a strand, ${\left(\varepsilon_{sh}\right)}_{t}$: shrinkage strain of concrete, $\Delta f_{pR}$: relaxation loss of a strand, n: modular ratio(=$E_{p}/E_{c}$), $E_{c}$: modulus of elasticity of concrete, $C_{t}$: creep coefficient, $f_{c}$: compressive stress of concrete at the location of a strand caused by prestressing, self-weight, and superimposed permanent dead loads, $A_{p}$: cross-sectional area of a strand, $A_{c}$: area of the concrete section, $I_{c}$: second moment of area of the concrete section, and $e_{p}$: eccentricity of the tendon centroid relative to the concrete centroid. More detailed analyses focused on the long-term PFs measured using Smart Strands, including a comparison with various design formulas, can be found in a previous study [17].

The theoretical PF showed good agreement with the measured PF when applying the exact $K_{T,smart}$, as it exhibited a similar decreasing trend, which in turn validated the measurement of PFs using the Smart Strands, the proposed procedure for TC of FBGs, and the design formula in Eurocode 2. It can also be seen from Figure 12 that when an inaccurate $K_{T,smart}$ is applied for TC, whether there is over- or under-compensation, the variation of the PF can deviate to a considerable extent from the normal trend expected from a design formula.

## 6. Conclusions

Fiber optic sensors are a promising solution due to their improved accuracy and durability compared to other sensors, the representative of which is the fiber Bragg grating (FBG) sensor. Like other sensors, the strain measured at an FBG can be affected by temperature; therefore, the temperature effect that is not related to the mechanical strain should be compensated for or corrected in the long-term measurement subjected to ambient temperature variation. However, the temperature compensation (TC) for FBG was often performed using an approximate formula or procedure in many previous studies. Further, relatively fewer formulas have been proposed that are appropriate for the TC of the FBG packaged with a certain material for practical purposes. Therefore, this study proposed a reasonable procedure of the TC for the FBG embedded in a packaging material and in a host material. In particular, the TC of the recently developed Smart Strand with the FBGs encapsulated into a carbon fiber reinforced polymer (CFRP) core wire of the seven-wire strand was investigated in detail both analytically and experimentally.

Based on the results of this study, the following conclusions can be drawn:

1. The various formulas on the TC of FBG in previous studies were revisited and the validity of each formula was discussed. In particular, the formula for the mechanical strain of a host material, which can be applied when the FBG is embedded in a packaging material or host materials, was derived for confirmation by considering the strain compatibility based on perfectly composite behavior between the FBG and the surrounding materials. This also revealed that some simplified formulas used for the TC in previous studies did not have a sufficient theoretical basis and only depended on a few assumptions that were not and cannot be justified.
2. The reasonable formula for the TC of a Smart Strand—including the thermal sensitivity ($K_{T,smart}$ = 13.1 × 10^−6^/°C)—was proposed by considering the mutual restraint between a CFRP core wire and steel helical wires when deformed by temperature change. The procedure can be comprehensively explained by introducing a concept of the equivalent thermal expansion coefficient of the Smart Strand. The mechanical strain of the Smart Strand obtained in this way can be converted to prestressing force (PF). This can lead to a reliable spatial distribution and temporal variation of the PF in the long-term measurement of PSC structures subjected to seasonal and daily variations in ambient temperature.
3. The derived thermal sensitivity and the coefficients included in the formulas were validated by realizing the dummy sensing condition in the chamber test of various specimens in a temperature-controlled circumstance. It was identified that the longitudinal length of the Smart Strand should be sufficiently long to ensure the composite behavior between a core wire and helical wires, which was assumed in the derivation of $K_{T,smart}$. There was only a 4.6% difference in $K_{T,smart}$ between the theoretical and experimental values.
4. To examine the applicability of the derived TC procedure of the Smart Strand, a 20-m-long post-tensioned full-scale specimen was fabricated and exposed to ambient temperature for 318 days. By applying the proposed $K_{T,smart}$, the PF showed a normal trend of a gradual decrease with time according to the long-term losses of prestress, which showed good agreement with the theoretical PF based on Eurocode 2. However, the PF without the application of $K_{T,smart}$ or with inaccurate values of $K_{T,smart}$ exhibited an unrealistically fluctuating trend following the variation in ambient temperature. Therefore, it can be concluded that a reasonable TC—as proposed in this study—is indispensable for the application of the developed Smart Strand to reliable PF measurement for safety assessments and the maintenance of PSC structures, such as in structural health monitoring.
5. Although this study focused on the pointwise FBG sensor, which is the most widely used fiber optic sensor, there are other types of fiber optic sensors, such as a Brillouin distributed sensor, that can realize spatially continuous measurement. Future research should extend the theories proposed in the current work by investigating the TCs of such distributed sensors.

## Figures and Tables

**Table 1:** Comparison of theoretical thermal sensitivity of a Smart Strand ($K_{T,smart}$).

Classification	Formula	Value ^1^ (×10^−6^/°C)	Error (%)	Remarks
Exact	$\alpha_{f}+\xi +\left(1-p_{e}\right)\left(\alpha_{smart}-\alpha_{f}\right)$	13.1	-	Equation (12)
Approximate 1	$\xi +\left(1-p_{e}\right)\alpha_{smart}$	13.0	0.8	$\alpha_{f}$ ignored.
Approximate 2	$\alpha_{h}+\xi$	16.2	23.7	$\alpha_{f}$ ignored, $\alpha_{smart}\approx \alpha_{h}$ assumed, and $1-p_{e}\approx 1$ assumed.

^1^ The following values of the coefficients were used for calculation: $\alpha_{f}$ = 0.5 × 10^−6^/°C, ξ = 6.2 × 10^−6^/°C, $p_{e}$ = 0.22, $\alpha_{smart}$ = 8.68 × 10^−6^/°C, and $\alpha_{h}$ = 10 × 10^−6^/°C (concrete).

**Table 2:** Comparison of various theoretical and experimental values of thermal sensitivity.

Classification	Thermal Sensitivity		Theory ^1^ (×10^−6^/°C)	Experiment (×10^−6^/°C)	
	Notation	Formula		Previous Test [31]	This Test
Fiber optic sensor (FBG)	$K_{T,f}$	$\alpha_{f}+\xi$	6.7	5.9	6.4
Fiber optic sensor (FBG) + CFRP core wire	$K_{T,f+c}$	$\alpha_{f}+\xi +\left(1-p_{e}\right)\left(\alpha_{c}-\alpha_{f}\right)$	6.3	5.5	5.5
Smart Strand: Fiber optic sensor (FBG) + CFRP core wire + steel helical wires	$K_{T,smart}$ or $K_{T,f+c+h}$	$\alpha_{f}+\xi +\left(1-p_{e}\right)\left(\alpha_{smart}-\alpha_{f}\right)$	13.1	12.5	6.6

^1^ The following values of the coefficients were used for calculation: $\alpha_{f}$ = 0.5 × 10^−6^/°C, ξ = 6.2 × 10^−6^/°C, $p_{e}$ = 0.22, $\alpha_{c}$ = 0, and $\alpha_{smart}$ = 8.68 × 10^−6^/°C.

## Data Availability

Not applicable.
